# Diagnosis and Management of Bacterial Meningitis in the Paediatric Population: A Review

**DOI:** 10.1155/2012/320309

**Published:** 2012-09-20

**Authors:** Catherine L. Tacon, Oliver Flower

**Affiliations:** ^1^Sydney Children's Hospital, Randwick, NSW, Australia; ^2^Intensive Care Unit, Royal North Shore Hospital, St Leonards, NSW 2065, Australia

## Abstract

Paediatric bacterial meningitis is a neurological emergency which, despite advances in medical management, still has a significant morbidity and mortality. Over recent decades new vaccines have led to a change in epidemiology of the disease; however, it remains a condition that requires a high index of suspicion, prompt diagnosis, and early management in the emergency department. New laboratory techniques and clinical tools are aiding the diagnosis of bacterial meningitis, yet some controversies still exist in its management. This paper outlines the changing epidemiology of the disease, current diagnostic techniques as well as controversies and advances in the management of bacterial meningitis in the paediatric population.

## 1. Introduction

Bacterial meningitis is a medical emergency characterised by inflammation of the meninges in response to bacterial infection. Untreated, its mortality approaches 100%, and even with current antibiotics and advanced paediatric intensive care, the mortality rate of the disease is approximately 5–10% [1]. Worldwide, the risk of neurological sequelae in survivors following hospital discharge approaches 20% [2]. Early diagnosis and appropriate management of the child with meningitis is therefore critical. The management and epidemiology of bacterial meningitis in the neonate differs from that of the infant and child; it will not be reviewed here.

## 2. Epidemiology

The incidence of bacterial meningitis is approximately 5–7 per 100 000 population [1]. In developed countries, *Neisseria meningitidis* and *Streptococcus pneumoniae* are now the commonest causes of acute bacterial meningitis in otherwise healthy children [3] (see Table 1). Previously, *Haemophilus influenzae* type B (Hib) accounted for up to 48% of all bacterial meningitis cases [4]; however, the introduction of the Hib vaccination program led to a dramatic reduction in the incidence of Hib meningitis. Hib now accounts for only 7% of meningitis cases in the United States and is predominantly seen in unvaccinated adult patients [4]. However, the burden of Hib in developing countries without adequate vaccination programs still remains significant; by 2007 only 42% of children worldwide had access to the Hib immunisation program [4].

*Streptococcus pneumoniae* is now the commonest cause of bacterial meningitis in the United States and Europe [4]. Although seen in the healthy child, children with a basilar skull or cribriform fracture with a CSF leak, asplenism or HIV infection are at particular risk of developing pneumococcal meningitis [3]. Furthermore, patients with cochlear implants have a 30 times increased risk of developing pneumococcal meningitis [5]. The development of pneumococcal conjugate vaccines has led to a decline in the incidence of pneumococcal meningitis in countries with an active immunisation program; however, concern exists as to the emergence of pneumococcal serotypes not covered by the vaccines [4]. This, coupled by the increasing resistance of *Streptococcus pneumoniae* to conventional antibiotics, is of growing concern [1].

There are six serogroups of *Neisseria menigitidis* with the ability to cause severe meningitis: A, B, C, X, Y and W-135 [6]. Infection with *Neisseria meningitidis* can be either epidemic or endemic [3], and although the majority of cases in the United States are sporadic [4], *N. meningitidis* is the only bacteria that can cause epidemics of meningitis [6]. Throughout America and Europe serogroups B, C, and Y account for the majority of meningococcal meningitis cases [4], with serogroup B being the leading cause of endemic meningitis in developed countries overall [6, 7]. Serogroup A *N. meningitidis* is also a significant problem, particularly in the sub-Saharan “meningitis-belt,” where it is responsible for a number of large-scale epidemics [6]. While a conjugate meningococcal vaccine for serogroups A, C, Y, and W-135 has shown reductions in meningococcal disease in some populations [3], development of an effective vaccine against serogroup B has been difficult. Recent trials have shown promise in the use of a new multicomponent serogroup B vaccine [7, 8], but currently the lack of a widely available, effective vaccination against *N. meningitidis* B, as well as the lack of access to vaccinations in populations at risk of epidemics, such as in sub-Saharan Africa, means that *N. meningitidis* still remains a significant cause of bacterial meningitis [6].

In developed countries less than 20% of bacterial meningitis in the paediatric population aged 3 months and over is caused by organisms other than *S. pneumoniae *or *N. meningitidis. *The less-common causative organisms include Group B *Streptococcus*, *Escherichia coli*, nontypeable *H. influenzae*, other gram-negative bacilli, *Listeria monocytogenes*, and group A streptococci [4]. In addition patients who have had penetrating head trauma or neurosurgery are also at risk of developing meningitis from staphylococcal species, streptococci, and aerobic gram-negative bacilli [3, 9], and this should be considered in such a child presenting with possible bacterial meningitis.

## 3. Diagnosis

Early diagnosis and treatment of bacterial meningitis is critical, and a high index of clinical suspicion is essential. Diagnosis involves both clinical assessment and the use of laboratory investigations.

### 3.1. Clinical

The clinical symptoms and signs of bacterial meningitis in children vary depending on the age of the child and duration of disease. Nonspecific signs include abnormal vital signs such as tachycardia and fever, poor feeding, irritability, lethargy, and vomiting [4]. Signs of fulminant sepsis such as shock, disseminated intravascular coagulation (DIC), purpuric rash, and coma may be present and are more common in meningococcal meningitis [1]. These signs however are more likely to develop later in the course of the illness (median time between 13 and 22 hours) [10] whereas nonspecific, often overlooked symptoms, such as leg pain, may be present within 8 hours in more than 70% of children with meningococcal meningitis and should prompt further immediate evaluation [10, 11]. Classical signs of meningitis such as nuchal rigidity, bulging fontanelle, photophobia, and a positive Kernig's or Brudzinski's sign (more common in children older than 12 to 18 months) may also be present [3]. A recent systematic review found that the presence of meningeal signs increased the likelihood of the diagnosis of meningitis, and conversely their absence decreased the likelihood [12]; however, other studies have shown that no classical symptoms and signs of meningitis are able to distinguish accurately between children with or without meningitis [13], and so these signs should be interpreted with caution. 

Seizures may be present in 20–30% of children with bacterial meningitis, more commonly with *S. pneumoniae* and Hib infections than with *N. meningitidis* [3]. A recent study has suggested that the presence of complex seizures more than doubles the risk of meningitis [12]. Focal neurological signs may also be found, as may a reduced level of consciousness. Coma on presentation is associated with a worse prognosis than a child presenting with irritability or lethargy alone [3].

### 3.2. Laboratory Investigations

#### 3.2.1. Lumbar Puncture

Whilst a lumbar puncture (LP) is necessary for the definitive diagnosis of bacterial meningitis and should be performed where a clinical suspicion for meningitis exists, contraindications often preclude this investigation. These contraindications (see Table 2) include signs of raised intracranial pressure, such as an alteration in level of consciousness, papilloedema, prolonged seizures, or focal neurological signs, as well as coagulation disorders, cardiorespiratory instability, a history of immunosuppression, certain central nervous syndrome (CNS) conditions, or localised infection at the site of insertion of the lumbar puncture needle [1]. LP may be delayed until these contraindications no longer exist; however, administration of antibiotics and appropriate therapy should not be delayed if the LP cannot be performed immediately.

Initial analysis of the CSF should include microscopy with gram stain, culture and measurement of protein, and glucose levels. CSF findings suggestive of bacterial meningitis are outlined in Table 3. Typically the CSF white cell count (wcc) is >1000 cells/mm^3^ although it may not be elevated in the early phase of the infection [3], and the majority of white cells are polymorphonuclear (PMNs). CSF protein is typically elevated (100–200 mg/dL) and glucose low (CSF to serum ratio <0.4) [3]. In untreated bacterial meningitis the CSF gram stain may be positive in 80–90% of patients [3] and varies with both the CSF concentration of bacteria and type of bacteria [9]. The overall probability of obtaining a positive gram stain result increases 100 times by using a cytospin technique [14] (the use of a high-speed centrifuge to concentrate cells). Patients with bacterial meningitis who have been pretreated with antibiotics are more likely to have a higher glucose and lower protein level although the CSF wcc and absolute PMN count are not normally significantly affected [15].

A clinical prediction rule, the Bacterial Meningitis Score, has been developed to assess the risk of bacterial meningitis in patients with CSF pleocytosis. It assesses patients as being of very low risk of bacterial meningitis if none of the following are present: positive CSF gram stain, CSF absolute PMN count ≥1000 cells/mm^3^, CSF protein ≥80 mg/dL, peripheral blood absolute PMN count ≥10 000 cells/mm^3^, and history of seizure before, or at the time of presentation [17]. The score however is not applicable to children with features of severe sepsis, known neurosurgical disease, known immunosuppression, traumatic lumbar puncture, or previous antibiotic therapy within the past 48 hours [18]. While a large multicentre study has validated this score, showing that if all criteria are absent, the risk of bacterial meningitis is 0.1% [17], as the score has less than 100% sensitivity, its use alone to decide individual patient therapy is not currently recommended [9, 18].

While the presence of an organism on gram stain, or culture of bacteria from the CSF, is diagnostic of bacterial meningitis, a number of other investigations may also be performed on CSF to aid diagnosis. Latex agglutination may be performed to detect the presence of bacterial antigens in the CSF. It has the advantage of being able to be rapidly performed, with a result available in less than 15 minutes, well before culture results are available [9, 19]. Although it may remain positive for up to 10 days after the initiation of antibiotics [19], it is neither 100% sensitive or specific [9, 19]. One study has shown a sensitivity of only 7% for detecting bacterial antigens in culture-negative bacterial meningitis [20]; hence, its use may be limited [4]. 

Polymerase chain reaction (PCR) may also be used to detect microbial DNA in CSF. It also has the advantage of being relatively rapid and is able to detect low amounts of bacteria in the CSF [21]. PCR results may be positive despite pre-treatment with antibiotics [21], and although not 100% specific, some studies have found PCR to have 100% sensitivity, allowing antibiotics to be ceased if PCR is negative [9], although further refinements in PCR techniques are probably necessary.

CSF lactate may be elevated in patients with bacterial compared with viral meningitis. Two recent meta-analyses have suggested that an elevated CSF lactate is a good distinguishing marker of bacterial meningitis [22, 23]. However as it may be affected by a number of factors, including pre-treatment with antibiotics (reducing the level), seizures, or cerebral hypoxia (increasing the level), its routine use in the assessment of community-acquired meningitis is not currently recommended, and further prospective studies are needed [9]. 

#### 3.2.2. Other Laboratory Investigations

Initial blood tests should be performed for full blood count, coagulation studies, and electrolytes to assess for complications of sepsis and to guide fluid management. Serum glucose should be routinely measured as it may be low in the child with meningitis, contributing to seizures. Its measurement is also needed to accurately interpret the CSF glucose.

Blood cultures should be performed in all patients with suspected bacterial meningitis. They may be of particular value if a lumbar puncture is contraindicated. The likelihood of a positive blood culture result varies with the infecting organism; 40% of children with meningococcal meningitis will have a positive blood culture, whereas 50–90% of *H. influenzae* and 75% of *S. pneumonia* meningitis patients will have a positive culture result [4].

Both CRP and procalcitonin have been evaluated to distinguish between viral and bacterial meningitis. Several studies have shown procalcitonin to have a better diagnostic accuracy than CRP in differentiating between aseptic and bacterial meningitis [24, 25]. Procalcitonin levels in combination with other clinical scoring systems have also been studied to evaluate the risk of bacterial meningitis [18, 26]. Although potentially increasing the sensitivity of scoring systems, the use of procalcitonin in association with clinical scores to exclude the diagnosis of bacterial meningitis is not currently recommended. As such, while an elevation in either CRP or procalcitonin is more suggestive of bacterial infection, neither can establish, nor exclude the diagnosis of bacterial meningitis [4, 24].

PCR for bacteria may be performed on blood and urine, especially if CSF is not obtainable.

Investigations are summarised in Table 4. 

### 3.3. Imaging

Computed tomography (CT) of the head is indicated if a child has signs of focal neurology, increased intracranial pressure (including papilloedema) deteriorating neurological function (such as increasing obtundation or seizures), immunocompromise or history of neurosurgical procedures, and shunt or hydrocephalus [1, 9] (see Table 2). In these patients it should be performed before a lumbar puncture is attempted although a normal CT scan does not entirely exclude the risk of raised intracranial pressure [1]. 

## 4. Management

**Figure 1:**
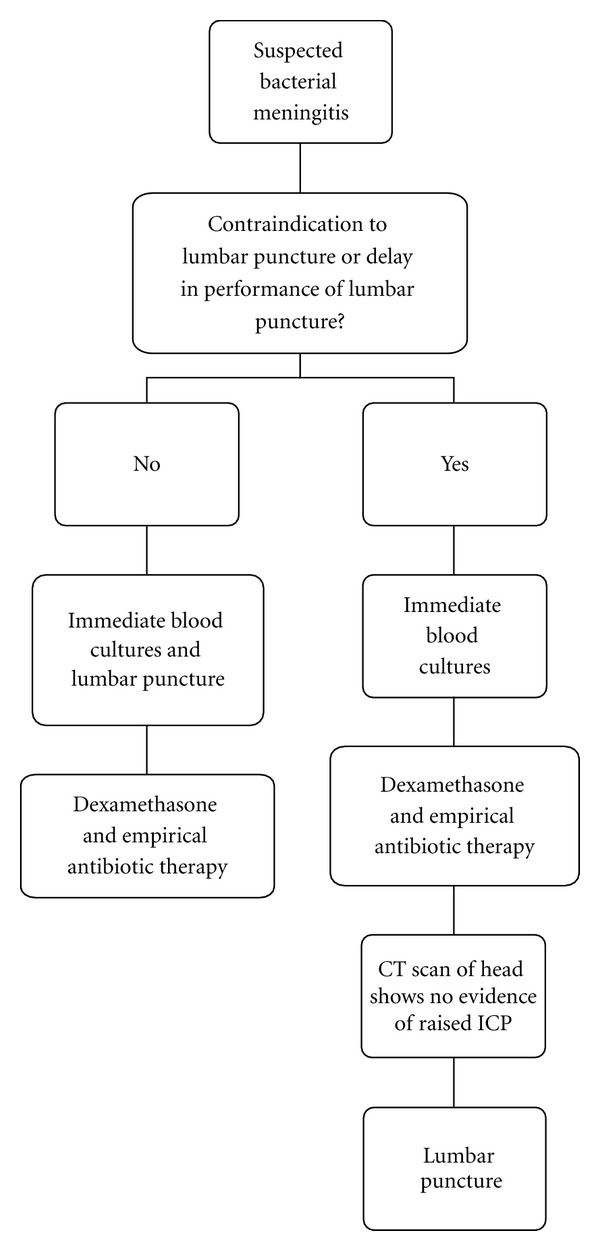
Management of suspected bacterial meningitis [9].

Bacterial meningitis is a neurological emergency, and it is critical that appropriate empirical antibiotics are administered as soon as possible after the diagnosis is considered. A flow chart for the management of suspected bacterial meningitis is provided in Figure 1.

### 4.1. Specific Therapy

#### 4.1.1. Antibiotics

The choice of empirical antibiotics is guided by knowledge of local resistance patterns of pathogens. Antibiotics should be administered parenterally, preferably by the intravenous route. In patients where intravenous access is not immediately possible, antibiotic administration should not be delayed, but given by the intraosseous or intramuscular routes. Most treatment guidelines recommend the use of a third-generation cephalosporin (such as ceftriaxone or cefotaxime) in conjunction with vancomycin as initial antibiotic therapy [9, 27]. Cefotaxime and ceftriaxone have excellent activity against all Hib and *N. meningitidis* strains. Increasing resistance of *S. pneumoniae* to penicillins has been reported, and although cefotaxime and ceftriaxone remain active against many penicillin-resistant pneumococcal strains, treatment failure has been reported [3], hence the addition of empirical vancomycin. *Listeria monocytogenes* is an unlikely pathogen in the immunocompetent child older than 3 months of age although the addition of benzylpenicillin to cover this organism may be considered for the immunocompromised patient [27].

Once the organism is isolated and sensitivities are confirmed, antibiotics may be rationalised. The duration of antibiotics is based primarily on expert opinion, rather than evidence-based data, and, although dependent on clinical response, common guidelines suggest a 7-day treatment course for Hib or *N. meningitides* and a 10–14-day course for *S. pneumoniae* [9]. A recent multicentre trial found that children with *H. influenzae*,* S. pneumonia,* or *N. meningitidis* meningitis could have antibiotics safely discontinued at 5 days, rather than 10 days if they were clinically stable [28]; this however has not been adopted as the current standard of care in most centres.

#### 4.1.2. Steroids

Empirical use of adjuvant dexamethasone (0.15 mg/kg/dose, 4 times a day) given before or up to a maximum of 12 hours after the first dose of antibiotics and continued for 2 to 4 days is currently recommended [9, 27, 29, 30]. This is based on evidence from studies in the late 1980s and 1990s that suggested improved neurological outcomes, particularly in hearing impairment, in children who had *H. influenzae* meningitis [9]. Recent studies have suggested that, unlike adults with bacterial meningitis, steroids do not improve mortality in children [31], and, hence, with the decline in incidence of Hib meningitis, the use of steroids in children with bacterial meningitis has increasingly been questioned. 

The most recent Cochrane review of the use of steroids in bacterial meningitis showed a significant reduction in hearing loss (from 20.1% to 13.6%) and severe hearing loss (from 11.2% to 7.3%) in children with meningitis, but no benefit on mortality [32]. Although overall this hearing benefit was seen in children affected by Hib meningitis, a subgroup analysis of children in high-income countries also showed a protective effect of steroids on hearing loss in non-*H. influenzae *meningitis [32]. This was not seen in low-income countries, in fact, overall no significant benefit of corticosteroids at all was found in children in low-income countries [32]. Other recent meta-analyses have found no benefit in any subgroup of children receiving adjuvant dexamethasone [33]. Overall, despite theoretical harmful effects of corticosteroids, no meta-analyses have shown harm with their administration, and as such it is still recommended to administer steroids before, or with the first dose of antibiotics, especially in the child with suspected Hib meningitis. Adjuvant dexamethasone should not be given to children who have already received antibiotics, as this is unlikely to improve outcome [9]. As dexamethasone has better penetration into the CSF than other corticosteroids, it is considered to be the corticosteroid of choice.

#### 4.1.3. Controversial Therapy: Glycerol

The use of oral adjuvant glycerol may be beneficial for children with bacterial meningitis through its action in increasing plasma osmolality, without inducing diuresis, leading to a reduction in cerebral oedema and an improvement in cerebral circulation and brain oxygenation [34]. A large randomised trial in Latin America showed a significant reduction in neurological sequelae in children given adjuvant glycerol, or glycerol in combination with dexamethasone, as compared with placebo [35]. No reduction in mortality or hearing impairment was seen [35, 36]. As glycerol is a relatively safe, cheap medication that can be administered orally, it may be especially beneficial in resource-limited settings. Some criticism however has been made about this large trial's design. This, in addition to a recent trial which failed to show any benefit of glycerol in adult meningitis patients [37], means that further well-designed prospective studies should be performed before glycerol is recommended as routine therapy. 

### 4.2. Supportive Care

Any child with a diagnosis of bacterial meningitis will need supportive therapy, which may include cardiorespiratory support in a paediatric intensive care unit and directed management of complications, such as seizures, cerebral oedema, SIADH, DIC, or shock. Early, protocolized, aggressive care by a consultant supervised paediatric team improves survival and outcomes [38]. Early intubation and ventilation should be considered for any child with evidence of respiratory compromise, threatened airway, ongoing shock, retractable seizures, or elevated intracranial pressure [30]. A recent Cochrane meta-analysis found some evidence to support the use of maintenance, rather than restrictive fluids in the first 48 hours [39]. This meta-analysis found an improvement in the rate of early spasticity and seizures and in later overall neurological sequelae in children receiving maintenance fluids [39]. These findings were however based on studies where late presentation and high mortality rates were common. In areas where early presentation is more common there are currently insufficient studies to definitively guide fluid management [39]. 

### 4.3. Chemoprophylaxis

Close contacts of all children with meningococcal meningitis should receive chemoprophylaxis (ceftriaxone, rifampicin, or ciprofloxacin), and contacts of those with Hib should receive ceftriaxone or rifampicin [3, 27]. Unvaccinated children less than 5 years of age should also be vaccinated against *H. influenzae* as soon as possible [27]. Patients should be kept in respiratory isolation for at least the first 24 hours after commencing antibiotic therapy [1].

## 5. Conclusion

Paediatric bacterial meningitis is a medical emergency which requires a high index of clinical suspicion, prompt diagnosis, and early, aggressive protocolized management. New vaccination programs have led to a change in epidemiology of the disease; however, it remains prevalent worldwide. Advances in clinical and investigation techniques are aiding the diagnosis of bacterial meningitis, and a combination of techniques is useful to confirm or exclude the diagnosis. While antibiotics, steroids, and supportive therapy remain the mainstay of treatment, further research should be performed into the roles of adjuvant therapy. 

## Figures and Tables

**Table 1:** Causative organisms.

Organism	Comment	
*Streptococcus pneumoniae*	Commonest organism Affects healthy children Additional risk factors: basilar skull or cribriform fracture, asplenism, HIV, and cochlear implants	

*Neisseria meningitidis*	Can cause epidemic, endemic, or sporadic infections	

*Haemophilus influenzae* type B	Reduced incidence after introduction of vaccination program	

Group B streptococcus	The less common pathogens Group B streptococcus, *E. Coli* and *L. monocytogenes* more common in neonates	
*Escherichia coli*		
Non typeable *H. influenzae *		
Other gram-negative bacilli		
*Listeria monocytogenes*		
Group A streptococci		

Staphylococcal species	Penetrating head trauma and neurosurgery	
Streptococci		
Aerobic gram-negative bacilli		

**Table 2:** Contraindications to lumbar puncture [9].

Contraindication	Comment
Raised intracranial pressure:	
Alteration in level of consciousness	
Papilloedema	
Focal neurological signs	Excluding an isolated cranial nerve VI or VII palsy
Prolonged seizures	Delay lumbar puncture for 30 minutes in simple, short seizures only

History of selected CNS disease	CSF shunts, hydrocephalus, trauma, post neurosurgery, or known space-occupying lesion

Immunocompromise	HIV/AIDS, on immunosuppressive therapy, post-transplantation

Coagulation disorders	

Cardiorespiratory insufficiency	

Localised infection at site of needle insertion	

**Table 3:** Lumbar puncture findings^1^ [3, 9].

CSF finding	Normal^2^	Viral	Bacterial	Partially treated bacterial
White cell count (cells/mm^3^)	<5	<1000	>1000	>1000
PMNs	0	20–40%	>85–90%	>80%
Protein (mg/dL)	<40	Normal or <100	>100–200	60–100+
Glucose (mmol/L)	≥2.5	Normal	Undetectable–<2.2	<2.2
Blood to glucose ratio	≥0.6	Normal	<0.4	<0.4
Positive gram stain	—	—	75–90% (depending on organism)	55–70%
Positive culture	—	—	>70–85%	<85%

^1^Other investigations may also be performed on CSF to exclude nonbacterial causes of meningitis depending on the clinical scenario; including India Ink staining or antigen testing for *Cryptococcus neoformans*, Herpes simplex virus (HSV), cytomegalovirus (CMV) and enterovirus PCR.

^2 ^Values for paediatric patients >1 month of age; some values vary for neonates [16].

Neonates: white cell count may be higher (<20 in the form of lymphocytes); normally zero PMNs, however some studies have found up to 5% PMNs in neonates without meningitis.

Neonates: normal protein <100 mg/dL.

**Table 4:** Investigations for suspected bacterial meningitis.

Investigation	Comment	
Blood:		
Full blood count	Neutrophilia suggestive of bacterial infection	
Serum glucose	Often low; allows interpretation of CSF glucose	
Electrolytes, urea, and creatinine	To assess for complications and fluid management	
Coagulation studies	To assess for complications	
Blood cultures	Positive in 40–90% depending on organism	
Inflammatory markers	Elevation suggestive of bacterial infection; procalcitonin of more value; neither can establish nor exclude diagnosis	
CRP, procalcitonin		

CSF:		
Protein and glucose		
Microscopy, culture, and sensitivities	Gram stain: * S. pneumoniae*—gram +ve cocci * N. menigitidis*—gram −ve cocci * H. influenzae*—gram −ve rod	
Latex agglutination^1^	Rapid; not 100% specific or diagnostic	
PCR^2^	Rapid; good sensitivity, techniques improving	
Lactate	Routine use not currently recommended	

Imaging: Computed tomography of the head	Indicated for focal neurology, signs of increased intracranial pressure (ICP), deteriorating neurological function, previous neurosurgical procedures, or immunocompromised May show evidence of hydrocephalus, abscess, subdural empyema, or infarction Normal scan does not entirely exclude risk of raised ICP	

Other: PCR on blood or urine	Useful if CSF not obtainable	

^1^Latex agglutination depends on laboratory availability; including *N. meningitidis, S. pneumoniae, H. influenzae* type B, *Escherichia coli* and group B streptococci.

^2^PCR depends on laboratory availability; including *N. meningitidis*, *S. pneumoniae*, *H. influenzae* type b, *L. monocytogenes*, HSV, CMV, Enterovirus and *Mycobacterium tuberculosis.*
